# Palliative, hospice, and end-of-life care special issue introductory editorial

**DOI:** 10.1016/j.pecinn.2024.100314

**Published:** 2024-07-04

**Authors:** Emily L. Mroz, Jordan M. Alpert

**Affiliations:** a Section of Geriatrics, Department of Internal Medicine, Yale School of Medicine, New Haven, CT, USA; b Nell Hodgson Woodruff School of Nursing, Emory University, Atlanta, GA, USA; c Center for Value-Based Care Research, Cleveland Clinic, Cleveland, OH, USA

In the relatively brief period since a specialized focus on end-of-life healthcare first emerged, the breadth and depth of the field's discoveries, pursuits, and practices have rapidly expanded. Seminal events in the latter half of the 20th century, including the founding of hospice in 1967, and the designation of palliative care as a medical specialty by the World Health Organization in 1990, ushered in a strong and still growing wave of clinicians, researchers, and service providers eager to explore avenues for relieving suffering and improving quality of life for seriously ill patients, their caregivers, and close others. Over time, this movement has evolved its goals, growing beyond what most had imagined possible [1] with services that are more regularly integrated upstream to maximize benefits [2]. Areas of focus have broadened and diversified, and subspecialities have developed to explore effective pathways for tailored end-of-life care across all serious illness contexts [3].

Importantly, research from the past several decades demonstrated that leading innovations to improve patient-centered end-of-life care generally “work as intended”: palliative care and hospice are now accessed as a set of inpatient, outpatient, community, and home-based services and complementary, integrated clinical techniques with an ever-expanding reach. These approaches have transformed the end-of-life experience by increasing patient satisfaction with medical care [4,5], improving symptom control and quality of life [[6], [7], [8]], infusing patients' values into care decision-making [9,10], reducing unwanted hospitalizations [11], minimizing costs [[12], [13], [14]], and more.

Despite the wealth of evidence of the positive impact of patient-centered end-of-life care approaches, the field continues to face barriers to offering maximum benefit to patients, families, and close others. Persisting lack of public awareness, social stigma, and misconceptions associated with hospice and palliative care [[15], [16], [17]], inequities in access to palliative and hospice care [18,19], and insufficient end-of-life care training and resources for clinicians [20] stymie the progress of this vital set of care approaches. The field must contend with these issues, which have endured since the beginning, while also wrestling with emerging controversies (e.g., best practices in advance care planning, provision of Medical Aid in Dying [MAID] practices). As the field expands and demand steadily rises, leaders must also wrestle with maintaining fidelity to the central goals of palliative care and encouraging larger cohorts of medical professionals to matriculate. This growing list of challenges must be met with innovative research stemming from multidisciplinary perspectives.

In this special issue of *Patient Education and Counseling Innovation,* our objective was to highlight innovative empirical research that can propel the field forward, address some of the above-mentioned controversies, showcase productive multidisciplinarity, and amplify the voices of underserved communities. Articles included in this special issue span a variety of topics and make recommendations for care outcome measurement, care best practices, educational resource development, and policy changes. A total of 18 articles are included, focusing on five topic domains, as described below.

**Communication between patients and clinicians.** The largest set of articles in this special issue focused on understanding and improving communication between patients and clinicians in serious illness and end-of-life contexts via the development of conceptual models of communication, examination of communication approaches, and exploration of barriers to communication. Felber et al., tackled the goal of supporting clinicians to feel more confident when discussing patients' proximity to death by developing a communication model to facilitate such conversations. Zelenski et al. described a training intervention that helped clinicians describe the best and worst outcomes associated with dialysis among patients with life-limiting illnesses. Crowe et al., characterized the contexts in which providers engage in empathic self-disclosure during Dignity Therapy for cancer patients. Ayele et al. conducted a qualitative study of the experiences of patients living with Parkinson's disease and their care partners disclosing, or holding back, sensitive palliative care issues or concerns with neurology clinicians. Relatedly, McDarby et al., characterized the questions patients living with neurologic illness, and their caregivers, ask during outpatient palliative care appointments, and the responses offered by clinicians. Many of these articles explore specialization in palliative care communication approaches, considering best practices in communication across specific care contexts. The articles also demonstrate innovation by exploring new territories for supporting clinicians to communicate efficaciously in the particularly sensitive context of discussing hopes and fears for end-of-life.

**Training clinicians to navigate complex care contexts.** Another set of articles in this special issue focused on developing training for clinicians to navigate complex care contexts with patients experiencing serious medical situations to end-of-life. These articles focused on non-physician care providers' skills to improve patient experiences in highly emotional or decisionally complex contexts. Wittenberg et al., for example, assert that nurses have unique opportunities to lead goals-of-care communication with patients and provide an integrated review of existing research which undergirds their recommendations for nurse communication training. With a similarly ambitious goal, Varner-Perez et al. present a pilot study of a chaplain-led decision-making intervention to support pregnant people during in the context of threatened periviable delivery. Pesut et al. contend that nurses require guided preparation for MAID practice and present a web-based guide to support their reflection on, and involvement in, this practice. Finally, Eskola et al. describe the value of interprofessional (versus physician-only) team training for integrated palliative care communication and present a model of a rapid training workshop to promote these skills.

**Bringing greater visibility to the experiences of diverse groups.** A third set of articles focused on bringing greater visibility to the experiences of diverse groups of individuals living with serious illness and close others. These articles are not only innovative for their focus on marginalized or understudied groups; they also break new ground by providing evidence of novel approaches to offering important visibility (i.e., through poetry, through narratives). First, Bybee et al. describe participants' perspectives on an approach for returning study results to sexual and gender minority couples facing cancer. Taladay-Carter presents a qualitative analysis of bereavement narratives, illustrating the influence of access, or in-access, to appropriate and patient-centered end-of-life care on the stories family members carry with them from loss experiences. Finally, Varilek et al. qualitatively depict the barriers to American Indian and rural patients living with cancer accessing appropriate, patient-centered care in advanced cancer contexts. They argue for improved access to upstream palliative care services in these communities.

**Development of pragmatic tools.** Quality end-of-life care depends on the identification of patients with care needs and the appropriate measurement of vital outcomes. Another set of articles in this special issue presents innovative approaches to address both of these needs. Bennett et al. qualitatively explore caregivers' perceptions of a prototype mortality risk calculator tool, with a focus on its acceptability if deployed in community contexts and its value for fostering earlier discussions about healthcare goals. Relatedly, Hughes et al. present a systematic review of existing end-of-life care decision aids, describing the effectiveness of decision-aids across a variety of outcomes representing decision aid success. And finally, Ratzel et al. assessed the feasibility of implementing multiple instruments for clinical routine care of oncology patients.

**Increasing access to, and engagement in, advance care planning.** We were unsurprised, yet excited, to see a set of articles on the development of new pathways for advance care planning. Carter et al. first describe leveraging community organizations to facilitate advance care planning workshops to increase access outside of clinical contexts. Relatedly, Walsh et al. explore the usability of a web-based advance care planning tool, with special attention to including family and close others in the planning process. Finally, Golden et al. examine ways to address advance care planning informational needs among surrogate decision-makers of patients living with dementia. Each of these articles adds important considerations to ongoing conversations about advance care planning use, access, and efficacy.

The 18 articles featured in this special issue emphasize the many ways palliative, hospice, and end-of-life care shape the experiences of patients, caregivers, and close others experiencing serious illnesses. They also highlight the implications of introducing innovative methods into clinical care. In equal measure, this issue demonstrates that there is still much work to be done to fully integrate patient-centered end-of-life care as an accessible standard of care. End-of-life care is relevant across disease contexts, and resources are still needed across clinician and patient populations. Our special issue includes articles focused on patients with diseases such as dementia, Parkinson's, cancer, and neurologic disorders, with other articles relevant to any and all patients, close others, or clinicians grappling with end-of-life. As research on end-of-life care continues to evolve, we hope this special issue serves as an impetus to improve communication, enhance clinician training programs, emphasize the perspectives of diverse groups, and continue efforts to develop innovative, pragmatic, and engaging tools that better the end-of-life experience for all involved.

## Declaration of competing interest

None.
